# Dispersal of juvenile Barrow’s goldeneyes (*Bucephala islandica*) mirrors that of breeding adults

**DOI:** 10.1186/s40462-023-00423-z

**Published:** 2023-10-12

**Authors:** T. M. Forstner, W. S. Boyd, D. Esler, D. J. Green

**Affiliations:** 1 Centre for Wildlife Ecology, Department of Biological Sciences, Simon Fraser University, Burnaby, BC Canada; 2 Pacific Wildlife Research Centre, Wildlife Research Division, Science and Technology Branch, Environment & Climate Change Canada, Delta, BC Canada; 3 U.S. Geological Survey, Alaska Science Center, Anchorage, AK USA

**Keywords:** Sea duck, Annual cycle, Dispersal, Migration, Population structure

## Abstract

Barrow’s goldeneyes across western North America have been shown to have a high degree of subpopulation independence using several data types. However, evidence for structured populations based on mitochondrial DNA, band recoveries, and tracking of adults is discordant with evidence from autosomal DNA. We used satellite tracking data from both juveniles and adults marked on natal and breeding grounds, respectively, in British Columbia, Canada to evaluate the hypothesis that male-biased juvenile dispersal maintains genetic panmixia of Pacific Barrow’s goldeneyes otherwise structured by migratory movements and high winter and breeding site fidelity of adults. We found that juvenile males traveled to overwintering sites located within the range of the overwintering sites of juvenile females, adult males, and adult females. Juvenile males migrated at the same time, travelled the same distance when moving between natal and overwintering sites, and had the same winter dispersion as juvenile females. Although juveniles did not travel with attendant females, all juveniles overwintered within the wintering range of adults. We tracked some juveniles into the following spring/summer and even second winter. Prospecting juveniles of both sexes travelled from their wintering grounds to potential breeding sites in the proximity of Riske Creek and within the bounds of the breeding locations used by adults. Juveniles tracked for more than a year also showed relatively high winter site fidelity. Because Barrow’s goldeneyes pair on wintering grounds, our tracking data are not consistent with the hypothesis that male-biased juvenile dispersal explains the genetic structure in the mitochondrial DNA and panmixia in the autosomal DNA of Barrow’s goldeneye. We suggest that uncommon or episodic dispersal of males might be enough to homogenize autosomal DNA but is unlikely to influence demographic population structure relevant to contemporary population management.

**Supplementary Information:**

The online version contains supplementary material available at 10.1186/s40462-023-00423-z.

## Introduction

Movement and dispersal patterns of juveniles have consequences for individual fitness, demography, and the genetic structure of populations [1–3]. Understanding juvenile movement throughout the annual cycle consequently facilitates management of migratory animals [4, 5]. Juvenile movements may be similar to those of adults when migration routes are genetically determined, or offspring migrate with their parents [6]. However, juvenile movements may differ as a result of their inferior flight performance and naive navigational skills, competition with adults, and responses to varying conditions resulting from differences in migration timing [7].

Studies conducted over the last three decades have revealed both age and sex-related differences in many aspects of avian migration. Juveniles can have longer migration routes [8], migrate more slowly [9], use wind drift less effectively [10], fly for shorter stretches at a time [11], and spend more time at stopover sites [8] than adults. Furthermore, migratory performance of long-lived birds can continue to improve for many years (e.g., Black Kites *Milvus migrans* [12], Cory’s Shearwater *Calonectris borealis* [13]). Differing migration patterns by juveniles or subadults could lead to greater likelihood for dispersal, relative to adults with more set migration patterns.

Recent studies on movements of sea ducks, aided by advances in tracking technology, are part of a growing body of research that addresses the need for full annual cycle approaches to the study of animal movement [14]. Researchers have described sea duck migration routes [15, 16], migration phenology [17], and migratory connectivity [18, 19] and used tracking data to identify critical habitat at different times of the year [20–22]. Most studies, however, only track movement of adult sea ducks and few have described movement of juveniles (but see [23, 24] for Harlequin Ducks, *Histrionicus histrionicus*; [25] for Common Mergansers *Mergus merganser*; [26] for King Eiders *Somateria spectabilis*). As many waterfowl pair in winter, movement patterns of unpaired juveniles prior to and during this period influence the degree of genetic structuring among geographies [26–28] and have implications for the degree of demographic connection among subpopulations [29].

Barrow’s goldeneye (*Bucephala islandica*) is a medium sized sea duck that, in western North America, winters along the Pacific Coast, migrates inland to breed in tree cavities alongside interior freshwater lakes, and may perform an additional northward migration to moult on shallow productive northern lakes [19, 30]. Several data types have been applied to determine the degree of population structuring within the Pacific range of Barrow’s goldeneyes; however, these pose a dilemma, as not all data types lead to a consistent conclusion. Mitochondrial DNA haplotype frequencies were highly different between Barrow’s goldeneye samples collected in Alaska and British Columbia [31]. Similarly, band recovery data indicated no evidence of overlap in winter recoveries between birds marked in Alaska and British Columbia [31, 32]. Also, extensive telemetry data [19, 33] showed that Barrow’s goldeneye have high migratory connectivity and site fidelity through the annual cycle, bolstering the conclusion that populations have significant structuring across western North America. In contrast, autosomal markers provide no evidence of genetic structure [28], suggestive of a single, panmictic population. Because of the existing data demonstrating that adult Barrow’s goldeneye have high site fidelity to both breeding and wintering grounds [33, 34] Brown et al. [28] hypothesized that dispersal of juvenile males maintains genetic connectivity across the species’ range.

Here, we use data on movements of juvenile and adult Barrow’s goldeneye tracked from their natal and breeding site in Riske Creek, British Columbia to wintering grounds on the Pacific coast to evaluate the hypothesis that male-biased dispersal explains the discordant evidence for population structure in Barrow’s goldeneyes. We compare distances travelled between the natal and wintering sites and winter dispersion of juvenile males with those of juvenile females and adult Barrow’s goldeneyes. For the individuals tracked into the following spring and summer, we also compare the prospecting movements of juvenile males, juvenile females and adults. In addition, we describe timing of the annual movements of juveniles in comparison with those of adults. We test for age-class and sex differences and the specific prediction that juvenile males disperse more widely than other classes.

## Methods

### Satellite telemetry and data processing

We captured 60 hatch-year (HY), pre-fledging Barrow’s goldeneye (35 males, 25 females) and 60 after-hatch-year (AHY) breeding adults (48 males, 12 females) near Riske Creek, British Columbia (57° 07′ N, 122° 27′ W; 2006, 2008, 2011). We used a decoy and mist nets to capture territorial adult males in spring. We used kayaks to herd hens and their ducklings into drive traps during the brood-rearing period. The HY birds were almost fully grown but still flightless at ~ 6 weeks of age when captured and tagged. We recorded mass and wing chord of each bird and estimated sex and age class based on plumage [35], cloacal characteristics, bursal depth [36], and by comparing masses and wing sizes within each family cohort, under the assumption that males are larger at fledging than females [30].

An experienced wildlife veterinarian surgically implanted a satellite transmitter, known as a platform terminal transmitters (PTT; 26-38g Microwave Telemetry and Telonics transmitters) into the coelomic cavity of each goldeneye following standardized methods described in Mulcahy and Esler [37]. PTTs were programmed to transmit locations for two to six hours every three to four days. PTT data (latitude, longitude, location error index, date (calendar day), time, temperature (°C), and battery voltage) were obtained from the Argos location and data collection system within 24 h of a satellite receiving a transmission. The Argos system estimates locations by calculating the Doppler shift in transmission frequency received by National Oceanic and Atmospheric Administration (NOAA) satellites as they move relative to a PTT. Locations are assigned an accuracy class; 3, 2, 1 and 0 are location classes with an estimated accuracy of < 250 m, 250–500 m, 500–1500 m, and > 1500 m, respectively; A and B are auxiliary locations where accuracy is not estimated; and Z is an invalid location [38]. Accuracy of each location is based on the transmitter-to-satellite geometry during a satellite pass, number of satellites, number of transmissions received, and stability of the transmission frequency [38].

Argos data were uploaded to Movebank (www.movebank.org). We used the Douglas Argos Filter (DAF) to remove redundant data and unlikely point locations [39]. We first employed the DAF hybrid filter, with MAXREDUN set to 15 km, and retained the highest accuracy location for each duty cycle. We subsequently applied additional filtering criteria manually by removing (i) all data from birds where the transmitter failed, or the bird perished within 14 days of PTT implantation [29, 40] and (ii) locations transmitted after a bird had died [33]. The temporal gap between transmissions was filtered to less than 30 days. The interval between transmissions ranged from 0.7 to 29.2 days (median: 4.69 days, IQR1-3: 3.23–6.09 days).

### Defining stages of the annual cycle and determining the phenology of migration

The annual cycle for adult Barrow’s goldeneye can be characterized by a wintering stage on the coast, spring migration, a breeding stage on interior wetlands, moult migration, a flightless, remigial moulting stage on interior lakes, and fall migration [19]. Females that successfully raise a brood typically moult on or near their breeding ponds, whereas males and unsuccessful females travel farther north to moult. Juveniles do not undergo a remigial moult or breed in their first year but may visit potential breeding locations as a subadult [30]. Juvenile females can pair in their first year (median pairing age = 2 years) but do not reproduce until at least 2 years of age (median first breeding age = 3 years; [34]). The annual cycle for juvenile goldeneye can therefore be characterized by their natal site, fall migration, a wintering stage on the coast, spring migration, a prospecting stage where they may visit breeding sites, moult migration, and a remigial moulting stage at the start of their second year. Juveniles may skip the prospecting stage and move directly from their wintering grounds to a moulting site. Similarities in the annual cycle of adults and juveniles allowed us to assign signal locations to stages of the annual cycle using criteria previously developed for adults (Table 1).

**Table 1:** Criteria to assign telemetry data from Barrow’s goldeneyes to stages of the annual cycle

Natal or breeding site	Location within the Riske Creek study area
Juvenile fall migration	Starts with an unreversed > 20 km movement away from the natal site and ends with the arrival on the wintering grounds on the west coast when directional daily movements of > 100 km switch to non-directional daily movements of < 100 km
Moult migration	Starts with an unreversed > 20 km movement away from the breeding site and ends at a location where individuals are stationary (locations are < 1 km apart over land) for > 30 days
Moulting area	An area defined by a series of locations where individuals are stationary (locations are < 1 km apart) for > 30 days between July and November
Adult fall migration	Starts with a > 1 km movement away from the moulting site and ends on arrival at the coast when directional daily movements of > 100 km switch to non-directional daily movements between points < 100 km apart
Wintering area	An area defined by a series of locations on the coast with non-directional daily movements between points are < 100 km apart
Spring migration	Starts with the first unreversed > 100 km movement away from the coast and ends with the arrival at an interior wetland with non-directional daily movements between points are < 20 km apart
Breeding area	An area defined by a series of locations at an interior wetland between April and July with non-directional daily movements between points are < 20 km apart
Staging area	Areas defined by a series of locations during migrations where points are < 20 km apart for > 7 days

Having assigned signal locations to stages for each individual captured at Riske Creek (the natal site or breeding location), we identified the geographic centre of the subsequent moulting location, wintering location, and breeding location of adults and the subsequent wintering, prospecting, and moulting locations of juveniles by calculating the mean-centre centroids for each stage. We plotted spatial data using ArcGIS Pro version 2.4.3 (Environmental Systems Research Institute (ESRI), Inc. Redlands, California, USA, and used the “argosfilter” package in R [41] to measure the straight-line geodesic distances between consecutive centroids, between capture locations at Riske Creek and winter centroids, and between capture locations at Riske Creek and prospecting/breeding centroids the following year.

Following De la Cruz [16], we then calculated departure and arrival as the median date between the last signal located within the area defining one stage and the next signal located outside that area or the median date between the last signal before and the first signal after entering the area defining the next stage of the annual cycle. We estimated total length of stay during each stage of the annual cycle as the difference between the departure date and the arrival date at each location, plus 1 day. This extra day is to account for the fact that a bird could have been present both on the day of arrival and/or the day of departure [16, 18].

### Statistical analysis

We examined age and sex-class effects on the timing of migration between stages of the annual cycle, the length of stay at each stage, and the distances travelled between stages using a series of Mann–Whitney U-tests because these variables did not conform to assumptions of normality and/or homogeneity of variances and standard transformations failed to normalize the data. However, conclusions based on these non-parametric tests did not differ from those conducted using t-tests conducted ignoring the non-normal data distribution.

Pairwise distances between individuals were used to characterize dispersion on the wintering grounds. We estimated pairwise distances using the geodesic distance between the wintering centroid of each individual and all other individuals in the same age and sex class. We then compared the winter dispersion of male, female, juvenile, and adult goldeneyes using a series of permanova tests implemented with the “betadisper” function in the R package “vegan” [42]. Permanova is a non-parametric alternative to MANOVA that uses the observed distance matrix and random permutations of pairwise distances to test for differences in the centroid or dispersion of individuals within groups. Permanova is more powerful and less sensitive to heterogeneity of dispersion across groups than MANOVA [43, 44]. We report the results of the permutation test (permutations; *n* = 9999) examining the homogeneity of multivariate dispersions using pairwise distances of individuals within the different sex and age classes. All statistical analyses were conducted using R [41].

## Results

We tracked movement of 52 juvenile (30 males, 22 females) and 53 adult Barrow’s goldeneye (43 males, 10 females) (Table 2). Fifteen birds (12.5%) failed to provide data because they died or the transmitter failed within 14 days of surgery. Median transmitter life for tracked birds was 155 (range 20–1103 days). We tracked 48 adults (39 males, 9 females) to their moulting sites, 32 juveniles and 28 adults to their wintering grounds, and 9 juveniles and 15 adults to potential breeding grounds the following year. A total of 8 juveniles and 15 adults were tracked for more than one annual cycle.

**Table 2:** Numbers of Barrow’s goldeneyes caught at Riske Creek, British Columbia between 2006 and 2011

Year	Age	Number Marked		Number Retained	
		Male	Female	Male	Female
2006	Adult (AHY)	23	–	19	–
2007	Adult (AHY)	15	–	14	–
2008	Adult (AHY)	10	10	10	8
	Fledgling (HY)	12	10	9	7
2009	Fledgling (HY)	10	7	9	7
	Adult (AHY)	–	2	–	2
	Fledgling (HY)	13	8	12	8

Number retained are those tracked for > 14 days post surgery and subsequently used in analyses of movement and dispersal

### Movement and timing of events in the annual cycle of juvenile Barrow’s goldeneye

**Fig. 1:**
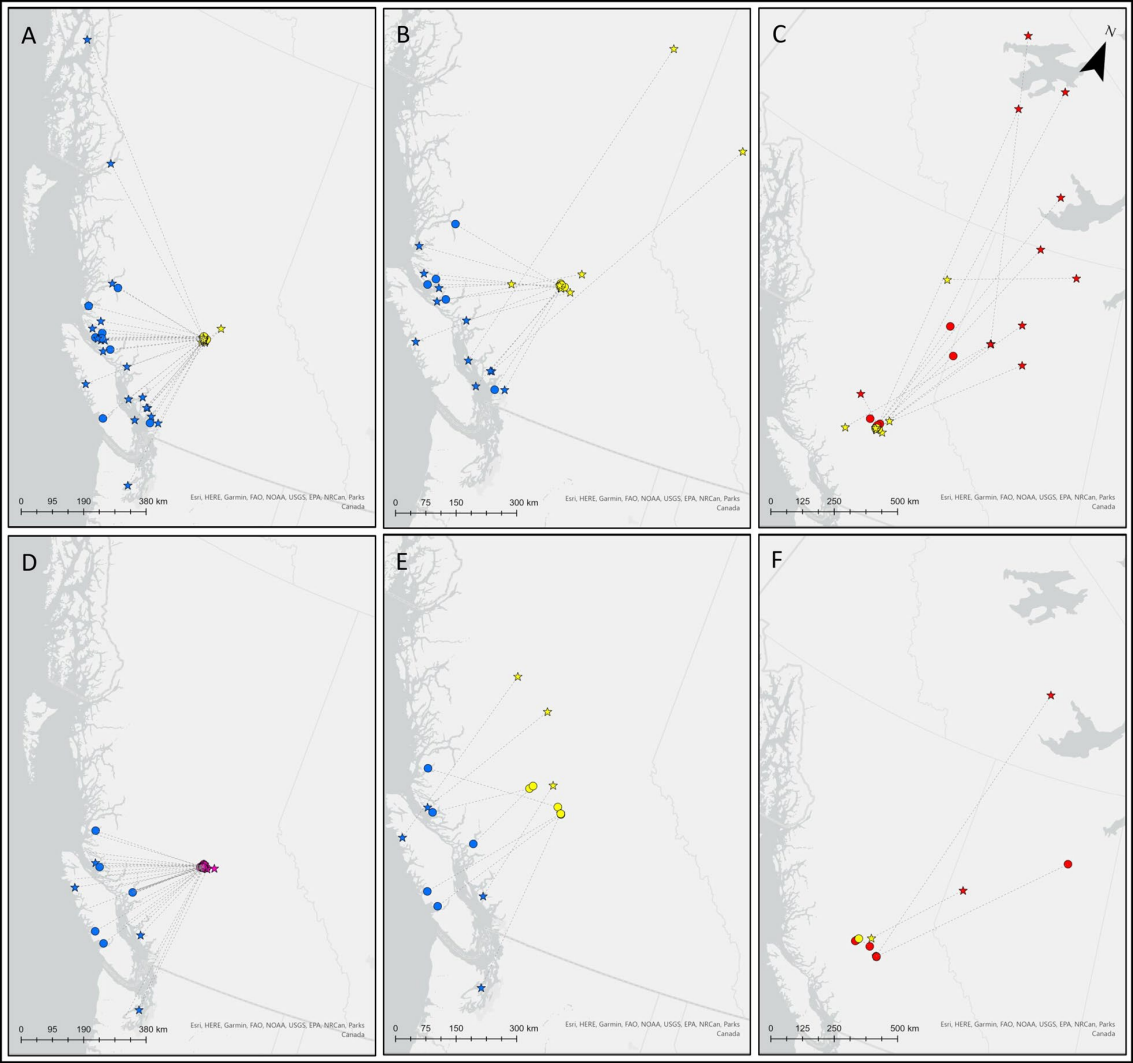
Movements of adult **A**–**C** and juvenile **D**–**F** Barrow’s goldeneye tracked with satellite transmitters deployed at Riske Creek, British Columbia. Panels A and C link the centre of individual breeding ranges (in yellow) or natal locations (in pink) with the centre of their wintering range (in blue). Adults do not move directly from their breeding to wintering range, but movement to the moulting grounds and from moulting grounds to wintering ranges are omitted. Panels B and E link the centre of individual wintering ranges (in blue) to potential breeding ranges (in yellow). Panels C and F link potential individual breeding ranges in the year after tags are deployed (in yellow) and centre of their moulting locations (in red). Females are represented with circles, males with stars. The number of points decreases from panels A-C and D-F as satellite transmitters fail or birds die

Juveniles left their natal area in Riske Creek over a 2.5-month period between August 13 and November 1. Juvenile males and females departed at approximately the same time (U = 240.5, *p* = 0.41, Table 3) with juvenile males leaving 3 months after adult males (U = 14, *p* < 0.001) and juvenile females leaving 2 months after female adults (U = 20, *p* < 0.001). After departing juvenile males and females made sporadic post-fledgling movements, exhibiting exploratory movements rather than utilization of distinct staging areas (n = 30, median = 32, IQ1-3 = 22–51, range = 6–82), before finally moving to the coast (Fig. 1). Juvenile males and females arrived on their wintering grounds at about the same time between October 1 and November 23 (males, median = November 2; females, median = November 1). (Table 3, U = 129.5, *p* = 0.59). Adult males and females typically took more direct routes to their moulting and wintering sites (Fig. 1) but having moulted, arrived on their wintering grounds at about the same time as juveniles (males, U = 208, *p* = 0.84; females, U = 93, *p* = 0.68, Table 3). Juveniles remained on their wintering grounds for more than 6 months, leaving between April 12 and June 7 (Table 3); males and female juveniles left their wintering grounds at the same time (U = 22, *p* = 0.35), approximately one month after adults (males, U = 8, *p* = 0.002; females, U = 0, *p* = 0.006).

**Table 3:** Summary of Barrow’s goldeneye arrival, departure, and length of stay between stages

Variable		Juvenile males				Juvenile females				Adult males				Adult females			
		Median	1st/3rd quartile	Range	Number of birds	Median	1st/3rd quartile	Range	Number of Birds	Median	1st/3rd quartile	Range	Number of birds	Median	1st/3rd quartile	Range	Number of BIRDS
Hatch or Breed	Depart	Sep 23	Sep 06/Oct 11	Aug 13–Nov 01	26	Oct 06	Sep 12/Oct 24	Aug 18–Nov 01	16	Jun 12	Jun 06/Jun 17	May 22–Jul 02	39	Aug 08	Aug 01/Aug 10	Jul 29–Aug 15	9
Moult	Arrive	N/A	N/A	N/A	N/A	N/A	N/A	N/A	N/A	Jul 01	Jun 18/10-Jul	Jun 08–Aug 01	39	Aug 09	Aug 05/Aug 12	Aug 01–Aug 16	9
	Depart	N/A	N/A	N/A	N/A	N/A	N/A	N/A	N/A	Oct 03	Sep 24/ Oct 13	Aug 29–Aug 01	25	Oct 06	Oct 01/Oct 14	Sep 20–Oct 28	8
	LOS (days)	N/A	N/A	N/A	N/A	N/A	N/A	N/A	N/A	92	75/113	55–154	25	61	57/66	51–74	8
Winter	Arrive	Nov 2	Oct 25/ Nov 05	Oct 01–Nov 23	21	Nov 01	Oct 29/Nov 03	Oct 24–Nov 07	11	Nov 01	Oct 28/Nov 05	Oct 12–Nov 10	20	Nov 03	Oct 29/Nov 07	Oct 25–Nov 14	8
	Depart	May 10	May 05/May 13	Apr 12–May 14	7	May 11	May 06/May 21	Apr 30–Jun 07	4	Apr 18	Apr 10/Apr 22	Mar 24–May 03	11	Apr 13	Apr 07/Apr 15	Mar 24–Apr 21	7
	LOS (days)	171	168/178	168–205	7	176	164/182	141–186	4	199	192/204	186–215	11	205	196/212	192–236	7
Breed	Arrive	May 14	May 11/May 24	May 07–May 25	5	May 27	May 09/Jun 14	May 07–Jun 14	4	Apr 30	Apr 19/May 08	Apr 11–Jun 12	9	Apr 17	Apr 15/Apr 18	Apr 09–Apr 22	6
	Depart	Jun 20	Jun 16/22-Jun	Jun 05–Jun 24	4	Jul 17	Jul 04/Aug 19	Jun 21–Sep 22	3	May 29	May 25/Jun 08	May 23–Jun 11	9	Jul 14	Jul 13/Jul 25	Jul 10–Aug 17	6
	LOS (days)	36	29/42	23–47	4	44	39/73	34–101	3	43	30/59	14–64	9	113	112/116	104–119	6
Moult	Arrive	Jul 17	Jul 04/Aug 21	Jun 11–Sep 23	4	Jul 18	Jul 05/Aug 21	Jun 22–Sep 23	3	Jun 23	Jun 08/Jul 12	May 25–Jul 17	9	Jul 25	Jul 16/Aug 09	Jul 11–Aug 23	6
	Depart	Oct 24	Oct 07/Oct 26	Oct 03–Oct 27	3	Oct 12	Sep 28/Oct 27	Sep 13–Nov 11	2	Oct 03	Sep 08/Oct 07	Jun 05–Oct 17	9	Oct 03	Sep 28/Oct 05	Sep 28–Oct 13	5
	LOS (days)	81	58/104	34–127	3	67	59/76	50–84	2	99	54/119	32–139	9	67	62/70	44–80	5

Medians, 1st/3rd quartiles, and ranges are presented

**Fig. 2:**
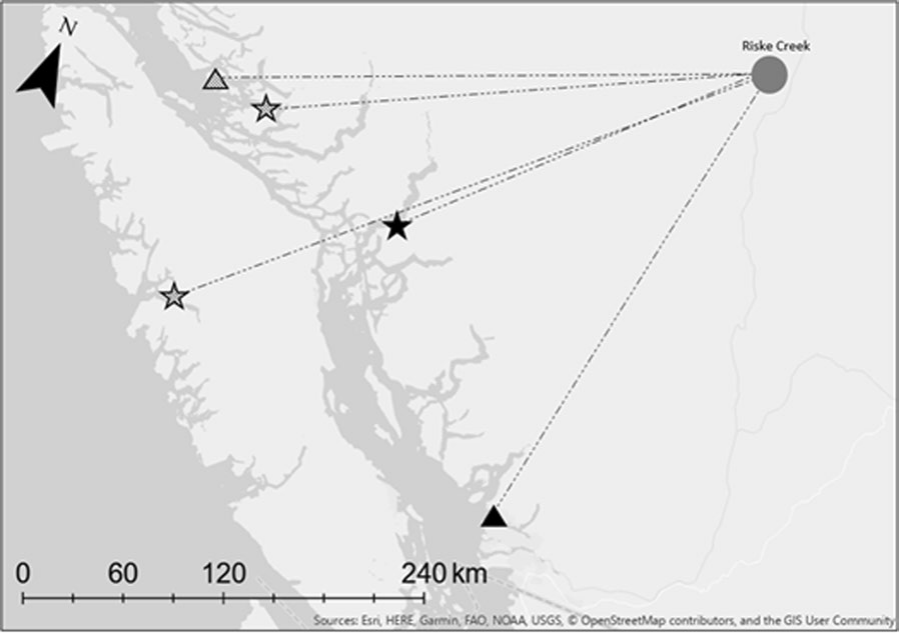
Mean-center centroids of two Barrow’s goldeneye families tracked with satellite transmitters. Dashed lines show direct-line movements of individuals from Riske Creek to wintering locations. The triangles represent adult females, and the stars represent their offspring from the same brood. The fill of the shapes (lined or solid) represent the two families

After fledging on natal areas, juveniles did not travel to the coast with attending females. In the two cases where juveniles and their attending adult females were tracked to their wintering grounds, juveniles remained at their natal site for 15–77 days after the female had departed and they occupied wintering areas 36 to 182 kms distant from their attending female (Fig. 2).

Juveniles of both sexes were observed to prospect, with 5 of 7 males and all 4 females that were still being tracked arriving at potential breeding sites at approximately the same time in late spring/early summer (U = 15.5, *p* = 0.52), 2–3 weeks after adults (males, U = 5.5, *p* = 0.007; females, U = 0, *p* = 0.006). Juvenile males and females remained at these sites for at least 1 month and departed at approximately the same time (length of stay, U = 9, *p* = 0.38; depart, U = 10, *p* = 0.22). Juvenile males departed later than adult males (U = 14, *p* = 0.09). Juvenile females left at the same time as adult females (U = 20, *p* = 0.83).

Tracking data in the year after tags were deployed was limited, but juveniles that continued transmitted to a subadult life stage, typically arrived at moulting sites between July and August of their second year and remained at these sites for 2–3 months, before departing in October (Table 3).

### Migration distances and dispersal of Barrow’s goldeneye

**Fig. 3:**
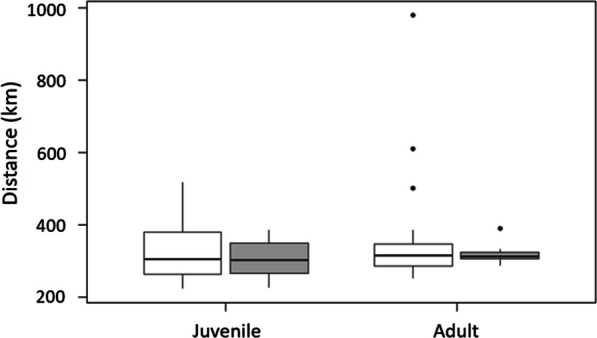
Migration distances between wintering and natal/breeding sites of Barrow’s goldeneye captured at Riske Creek, British Columbia between 2006 and 2009. Migration distances were calculated between mean-center centroids. White boxes represent males and grey boxes represent females

Juveniles captured at Riske Creek wintered along the coast between latitudes 47.72686° and 51.59177°, within the core wintering area used by adults (Fig. 1). Migration distances, measured as the straight-line distance between natal sites and wintering centroids of juvenile males and females did not differ (males, median = 308 km, range = 222–517 km; females, median = 283 km, range = 224–383 km; U = 105, *p* = 0.55). Migration distances for juveniles also did not differ from the straight-line distance between Riske Creek and wintering centroids of adults (males U = 244, *p* = 0.55; females U = 49, *p* = 0.37; Fig. 3).

**Fig. 4:**
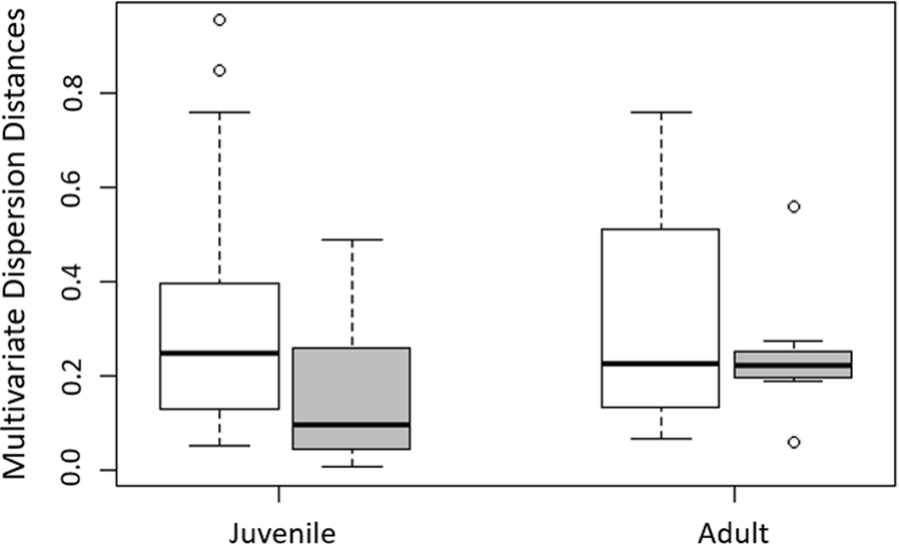
Multivariate dispersion between consecutive winters for juvenile and adult Barrow’s goldeneyes on the Pacific coast captured at Riske Creek, British Columbia between 2006 and 2009. Pairwise distances were calculated between mean-center centroids. White boxes represent males and grey boxes represent females

There was also no evidence that the winter distribution of juvenile males was more dispersed than the distribution of juvenile females or adult males (Fig. 4). Dispersion (i.e., variance in the distance from the spatial centre of the wintering locations of all individuals of the same age/sex-class) of juvenile males did not differ from the dispersion of juvenile females (*p* = 0.14), and dispersion of juvenile males was slightly lower than the dispersion of adult males (*p* = 0.98; Fig. 4). Dispersion of juvenile females did not differ from the dispersion of adult females (*p* = 0.37; Fig. 4).

Prospecting juveniles travelled from their wintering grounds to potential breeding sites in the proximity of Riske Creek and within the bounds of the breeding locations used by adults (Fig. 1). Migration distances for juvenile males and females did not differ (males, median = 380 km, range = 325–490 km; females, median = 291 km, range = 205–382 km; U = 6, *p* = 0.24). Migration distances of juveniles also did not differ from those of adults (adult male, median = 352 km, range = 179–918 km; adult female, median = 309 km, range = 301–389 km; males U = 16, *p* = 0.097; females U = 22, *p* = 0.83). Straight-line distances between natal sites and prospecting sites of juvenile males were greater than those of juvenile females, but the difference between sexes was not significant (male, median = 73 km, range = 1–355 km; female, median = 16 km, range = 1–96 km; U = 91, *p* = 0.34). However, straight line distances between natal and prospecting sites of juveniles differed from straight line distances between capture locations and subsequent breeding sites of adults (males, U = 31, *p* = 0.29; females, U = 3, *p* = 0.05).

Juveniles travelled between 2 and 1226 km from prospecting sites to moulting sites, where they would undergo their first remigial moult in their second year. Juvenile males migrated much farther than juvenile females (male, median = 837 km, range = 406–1226 km; female, median = 6 km, range = 2–53 km; U = 0, *p* = 0.80). However, moulting sites of juveniles did not extend as far north as those of adults (Fig. 1).

**Fig. 5:**
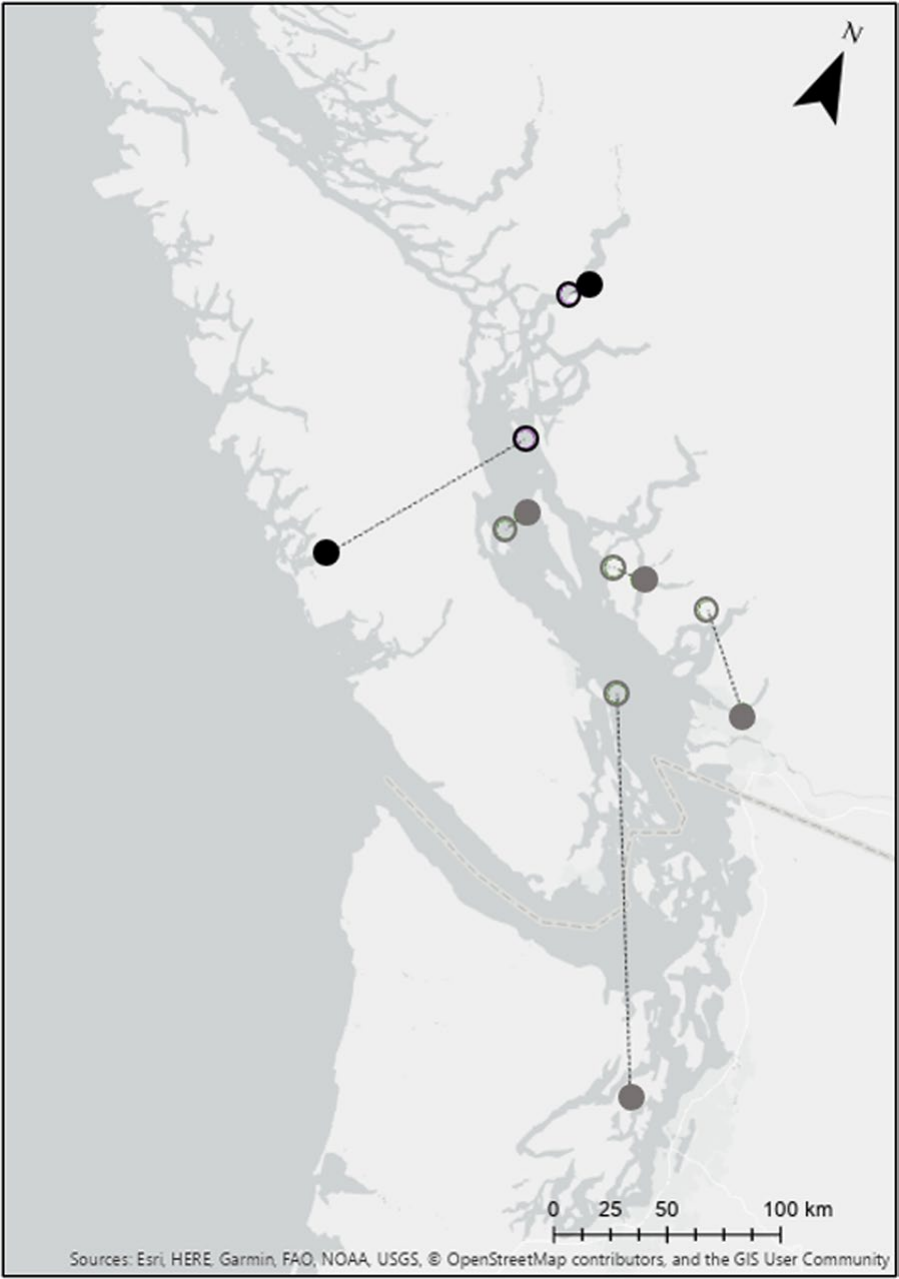
First and second winter mean-center centroids for juvenile Barrow’s goldeneye captured at Riske Creek, British Columbia between 2006 and 2009 and tracked with satellite transmitters. Males are dark grey and females are black. Solid circles represent wintering locations in the first winter and open circles represent the second winter

We were able to determine inter-annual winter site fidelity for 6 juveniles and 15 adults. Juvenile wintering locations were located a farther distance from the previous wintering location than adults’ consecutive wintering locations. Of the 6 juveniles that had tracking data for a second winter, 2 individuals (1 male and 1 female) wintered more than 100km from their first wintering location, 1 male individual wintered between 10 and 100km from the first wintering location, and 3 individuals (1 male and 2 females) wintered less than 10km from their first wintering location (Fig. 5). All of the 15 adults (10 males, 5 females) that had tracking data for a second winter, wintered less than 100 km from their first wintering location. Locations of wintering sites of adult males between consecutive winters were farther apart than those of adult females. Only 2 adult males moved more than 50 km from their first wintering locations, however, no females moved greater than 10 km from their first wintering location.

## Discussion

Age and sex-specific differences in movements of migratory birds are well documented. Compared to adults, juvenile movements between key stages of the annual life cycle may occur at different times [8, 11], at different speeds [9], and along different routes [8]. In this study we found that juvenile Barrow’s goldeneyes initially remained close to their natal areas while adults migrated north to moult and then juveniles followed a slow and indirect route to their coastal wintering grounds. They consequently arrived on their wintering grounds at approximately the same time and in the same area used by adults. Juveniles remained on their wintering grounds longer than adults but, like adults, left the coast in spring and returned to interior ponds and lakes near Riske Creek (their natal and adult breeding site). Contrary to a hypothesis of higher dispersal by juvenile males, movements of male and female juveniles were very similar. Juvenile males and females left their natal area at the same time, travelled a similar distance to overlapping wintering grounds and remained on the coast for approximately 6 months before prospecting for future breeding opportunities close to their natal origins. Differences in the movement, dispersion, and philopatry of juvenile males and females in their first year, therefore, do not provide an explanation for differences in the observed genetic structure of the autosomal and mitochondrial genome in this species.

Juveniles of many gregarious species are also thought to learn migration routes by travelling with experienced adults [45]. Juvenile movements may differ in timing from the majority of adults but nevertheless result in migration to shared wintering grounds if some adults remain and care for young and naïve young subsequently migrate with these experienced adults. Juvenile Barrow’s goldeneye that are abandoned by their parents are known to join other broods and form creches where a single female cares for multiple broods [32]. Byholm et al. [46] provided evidence for cultural inheritance of migration routes in Caspian terns while also showing that naïve young that migrated with a genetic or foster parent were more likely to survive their first migration than young that lost contact with their parent. However, we found little evidence to suggest that juvenile Barrow’s goldeneye migrate to the coast with attendant females. Juveniles, on average, left their natal territories 2 months after adult females and, in the cases where juveniles and their attendant females were both tracked, juveniles left 15–77 days after the adult female. In addition, juveniles and their attendant females were tracked to different over-wintering sites. Similarly, juvenile King eiders form creches but do not appear to migrate with their attendant females [26]. However, all juvenile Barrow’s goldeneye tracked in this study wintered along the stretch of coast (between central British Columbia and Hood Canal, Washington) where breeding adults from Riske Creek also winter. Juveniles tracked for more than one year then spent their second winter within 178 km of their first wintering site; this supports previous work documenting a high level of overwinter site fidelity in both adult and juvenile Barrow’s goldeneye [33]. Although our understanding of how migration patterns of sea ducks are transmitted from one generation to the next remains limited our study suggests that genetic inheritance and social attraction, such as the imprinting of mother ducks on their ducklings, likely play a more important role than social learning in Barrow’s goldeneyes.

Male-biased dispersal and female-biased natal philopatry are well documented among waterfowl [47, 48]. This is thought to provide greater benefits to females by returning to a familiar area and potentially breeding near their mother [49, 50]. Mark-resighting data from 811 ducklings marked at our 300 km^2^ study site near Riske Creek, British Columbia showed that female ducklings were six-times more likely than males to return and be resighted at the study site the following year [34]. However, few female ducklings and no male ducklings subsequently recruited to the breeding population at this site [34], but see 30]. Similarly in this study, all female ducklings tracked to a potential breeding site returned to the vicinity of Riske Creek (4 of 4 females visited breeding sites within 5 km of their natal site) whereas only 2 of 4 males returned to the vicinity of Riske Creek (0 visited breeding territories within 5 km of their natal site). Male ducklings that visited potential breeding sites were also tracked to areas slightly farther from Riske Creek. However, because median age of first breeding for females is estimated to be 3 years and for males to be 4 years [30], our tracking data cannot be used to estimate and compare the natal dispersal or natal philopatry of juvenile males and females.

Sex-biased dispersal has been posited as an explanation for differences in the genetic structure of the mitochondrial and autosomal genome of waterfowl, including Barrow’s goldeneye [e.g., 25, 28, 51. Simulation models suggest that male-biased dispersal is a better explanation for the greater population structure in mitochondrial DNA than the smaller effective population size and faster sorting rate of mitochondrial relative to nuclear DNA [52]. However, we found no evidence that sex-differences in movements of juvenile Barrow’s goldeneye in their first year explains the discordance between the structure of the mitochondrial and nuclear genome. Juvenile males and females travelled a similar distance between natal sites and their first overwintering area. Overwintering distributions and the dispersion of males were similar to those of females. In subsequent years, both male and female juveniles were faithful to these overwintering sites, where pairs form. However, the lack of support for the hypothesis that long-distance dispersal by juvenile males maintains genetic connectivity of Barrow’s goldeneye requires some caution. First, it is challenging to observe and quantify the long-distance dispersal events that could result in genetically panmictic populations using direct tracking methods because those movements are rare relative to short-distance dispersal events [53] and thus hard to detect. Second, it requires relatively few long-distance dispersal events per generation to maintain genetic connectivity across a species’ range [e.g., 54. Therefore, it is possible that rare movements by juvenile males do, in fact, explain the observed panmixia in autosomal DNA. However, it is important to recognize that rare movements likely do not have meaningful demographic effects across otherwise structured subpopulations, i.e., they do not markedly change abundance or trends of subpopulations, which are the attributes that are most relevant to contemporary population management [55].

Studies documenting juvenile movements can improve understanding of the dynamics and structure of bird populations but can also inform management activities and mitigation efforts for wildlife that have age- or sex-specific migration strategies [24, 25, 56, 57]. In this study we document some broad-scale differences between adult and juvenile Barrow’s goldeneye movements throughout the year. Adults breeding at Riske Creek, British Columbia, especially males, migrate up to 1698 km to moult on northern lakes in the Northwest Territories between May and August [19], whereas juveniles spend this time closer to their natal origins in interior lakes and ponds to the south. Similarly, although both adults and juveniles spend a significant proportion of the year at wintering sites along the coast, juveniles delay their spring departure to interior areas by approximately one month. As a result of differential migration timing by age class, age classes have different exposures to events and environmental conditions across different stages of the annual cycle. For example, because juveniles spend more time on nonbreeding areas, they have higher risk of exposure to marine-based contamination (e.g., oil spills) than adults. Also, adult Barrow’s goldeneyes have been shown to adjust their spring migration to accommodate variation in weather and snow conditions [58], which juveniles likely do not need to do given their later departure. Recognition of age-specific movement and habitat use may be important when considering environmental or anthropogenic effects on wildlife populations (Additional file 1).

### Supplementary Information

**Additional file 1. Figure 1A.** Representative tracks of female and male adults (1,2) and juveniles (3,4). Annual cycle stages are denoted by color (hatch = pink, breed = orange, moult = green, winter = blue).

## Data Availability

Argos PTT data used in this study are available from Movebank in the Data Repository file “Migration Patterns of Pacific Sea Ducks”. https://www.movebank.org/cms/webapp?gwt_fragment=page=studies,path=study1441422788
