# Supplementary material for: Dispersal of juvenile Barrow’s goldeneyes (Bucephala islandica) mirrors that of breeding adults
Source: Mov Ecol. 2023 Oct 12;11:62. doi: 10.1186/s40462-023-00423-z (PMC10568906; doi:10.1186/s40462-023-00423-z)
Supplement: Supplementary file 1 — Additional file 1. Figure 1A. Representative tracks of female and male adults (1,2) and juveniles (3,4). Annual cycle stages are denoted by color (hatch = pink, breed = orange, moult = green, winter = blue). [file 40462_2023_423_MOESM1_ESM.docx]

**Figure 1A.** Representative tracks of female and male adults (1,2) and juveniles (3,4).


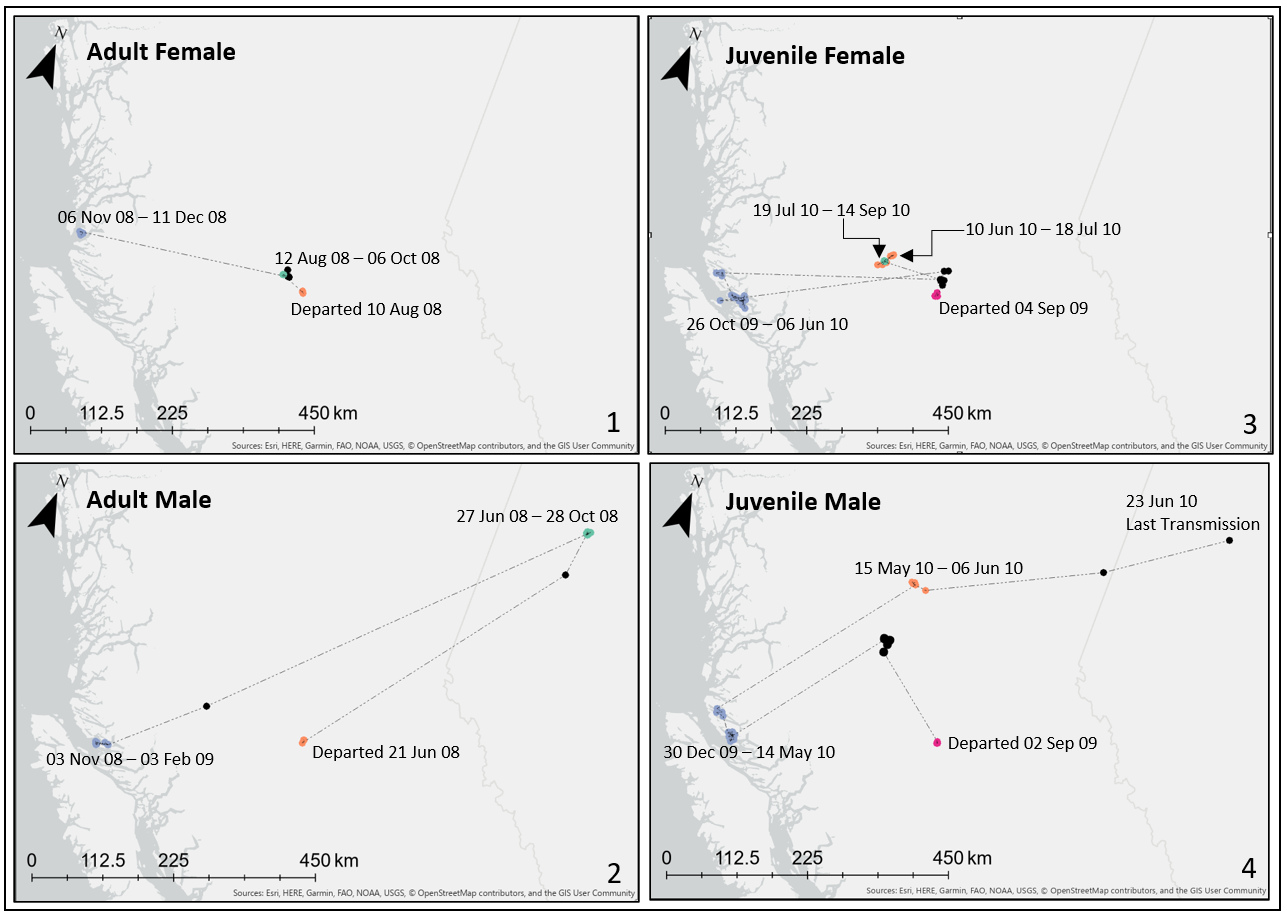


Annual cycle stages are denoted by color (hatch = pink, breed = orange, moult = green, winter = blue).
